# The expression of *SERPINE1* in colon cancer and its regulatory network and prognostic value

**DOI:** 10.1186/s12876-022-02625-y

**Published:** 2023-02-08

**Authors:** Yigang Wang, Jinyan Wang, Jianchao Gao, Mei Ding, Hua Li

**Affiliations:** 1 Anus and Intestine Surgery, Tangshan Central Hospital, Tangshan, 063000 Hebei China; 2 Department of Gastrointestinal Surgery, Tangshan Central Hospital, Tangshan Youyi Road and Changning Road Interchange Westbound 300 Meters, Tangshan, 063000 China

**Keywords:** *SERPINE1*, Bioinformatics, Colon cancer, Regulatory network, UALCAN, LinkedOmics

## Abstract

**Background:**

Serpin Peptidase Inhibitor 1 (*SERPINE1*) promotes cancer progression by making it easier for cancer cells to spread to surrounding normal tissue. We expect to understand the prognostic value and regulatory network of *SERPINE1* in colon cancer using bioinformatics methods.

**Methods:**

The expression of target gene *SERPINE1* in varying cancers was analyzed by the Tumor Immune Estimation Resource (TIMER) database. *SERPINE1* expression in Colon Adenocarcinoma and normal tissue samples was assessed by starBase and UALCAN databases. *SERPINE1* expression in clinical tissues was assayed using quantitative reverse transcription Polymerase Chain Reaction (qRT-PCR). *SERPINE1* expression was detected in colon cancer patients with various clinical features (age, gender, nodal metastasis status, race, stages, and subtype) using analysis of variance. Survival curve was used to analyze the effect of high and low expression of *SERPINE1* on the survival time of patients with different clinical phenotypes. Gene Set Enrichment Analysis (GSEA) was conducted on the results of LinkFinder calculation using LinkInterpreter module, which was combined with Pearson correlation analysis to obtain the kinase targets and miRNA targets, transcription factor targets, and corresponding signaling pathways associated with *SERPINE1*. The Gene Ontology (GO) and Kyoto Encyclopedia of Genes and Genomes (KEGG) were performed on GSEA result. Finally, Gene Multiple Association Network Integration Algorithm (GeneMANIA) was utilized to establish a network of genes related to the kinases *MAPK1*, *miR-18a*, and *SRF_Q*, and biological functions were analyzed.

**Results:**

Based on TIMER, starBase, and UALCAN databases, *SERPINE1* was found to be remarkably highly expressed in colon cancer patients, which was further verified by clinical tissue. It was also associated with different clinical features (nodal metastasis status, stages, subtypes). Additionally, survival analysis showed that patients with low expression of *SERPINE1* had a longer survival time, suggesting that *SERPINE1* was a prognostic risk factor for colon cancer. Pearson correlation analysis revealed that the expression of Integrin Alpha 5 (*ITGA5*), Matrix Metallopeptidase 19 (*MMP19*), and ADAM Metallopeptidase with Thrombospondin Type 1 Motif, 4 (*ADAMTS4*) had the highest correlation with that of *SERPINE1*. The GSEA results indicated that these genes were mainly enriched in the pathways of RNA expression and kinases. Finally, GeneMANIA analysis was introduced to construct the molecular network of *SERPINE1*.

**Conclusion:**

Overall, our bioinformatics analyses comprehensively described the networks involved *SERPINE1* in colon cancer and the potentially associated molecular mechanisms.

## Introduction

Colon cancer is a common malignancy that occurs in the gastrointestinal tract. According to statistics from the National Cancer Institute, as of the first three quarters of 2022, there were about 150,000 cases in the United States, and it caused about 50,000 deaths. Many risk factors lead to the onset of colon cancer: family history, gene mutations, disease history, excessive drinking, smoking, and obesity [1]. Existing studies found that gene mutation is a major risk factor for colon cancer. For instance, mutations in *K-RAS* can stimulate colon cancer, and mutations in *TP53* can promote early colon cancer deterioration and transformation into metastatic colon cancer [2]. Change in gene expression levels is also a key factor affecting the onset and prognosis of colon cancer. Numerous studies analyzed the gene transcriptional levels of colon cancer patients and found key genes such as *GLUT1*, *MUC2*, *Cyclooxygenase-2*, etc. that are related to the prognosis of colon cancer [3, 4]. Recent studies on colon cancer-related gene regulatory pathways found that MAPK signaling pathway, JAK/STAT, AKT/NF-κB and other signaling pathways can modulate the occurrence of colon cancer [5–7]. Therefore, we believed that the analysis and mining of colon cancer-related genes would deepen our understanding of colon cancer and improve colon cancer treatment.

Serpin Peptidase Inhibitor 1 (*SERPINE1*), also known as plasminogen activator inhibitor-1 (*PAI-1*), is an encoding gene of serine protease inhibitors that can suppress fibrinolysis *in vivo* [8]. *SERPINE1* is closely correlated with the morbidity of multiple cancers. Studies illustrated that *SERPINE1* can stimulate the progression of triple-negative breast cancer by promoting cytoskeletal reorganization and glycometabolism and can also accelerate the progression of pancreatic cancer by up-regulating the secretion and expression of *IL-8* [9, 10]. The role of *SERPINE1* in the pathogenesis of colon cancer was studied previously, and a current study found that *SERPINE1* can promote the progression of colon cancer through the P38-MAPK pathway [11]. Since the expression of *SERPINE1* is closely related to cancer, it is vital to understand the regulatory mechanism of *SERPINE1* expression in vivo. Current research believed that *miR-143* can reduce the expression of *SERPINE1*, thereby preventing the progression of bladder cancer [12]. It was also found that *SERPINE1* interacts with the membrane receptor LDL receptor-related protein 1 to enhance proliferation and migration of esophageal squamous carcinoma cells, and activate AKT and ERK signaling pathways [13]. Due to its complex regulatory effect in vivo, the study of *SERPINE1* and its regulatory mechanism will shed light on further understanding of pathogenesis and the early screening of colon cancer.

In this study, multiple databases (including Tumor Immune Estimation Resource (TIMER), starBase, UALCAN, and The Human Protein Atlas) were used to find that *SERPINE1* was markedly overexpressed in colon cancer, and *SERPINE1* expression was correlated with clinical features of colon cancer patients. Survival curve was used to analyze patient survival rate. In this study, survival curve was utilized to analyze the prognostic effect of *SERPINE1* on colon cancer, and it was found that patients with high *SERPINE1* expression had poor prognoses. LinkedOmics is a multi-omics analysis tool based on The Cancer Genome Atlas (TCGA) database. The highly correlated genes (Integrin Alpha 5 (*ITGA5*), Matrix Metallopeptidase 19 (*MMP19*), and ADAM Metallopeptidase with Thrombospondin Type 1 Motif, 4 (*ADAMTS4*)) of *SERPINE1* were obtained by Pearson correlation analysis. Gene set enrichment analysis (GSEA) was used to identify *SERPINE1*-associated kinases, miRNAs and transcription factors. Gene Multiple Association Network Integration Algorithm (GeneMANIA) is a simple co-expression gene analysis software that can be used to establish gene regulatory networks and analyze the biological functions of genes. In this study, GeneMANIA was utilized to obtain network based on *MAPK1*, *miR-18a* and *SRF-Q6* and the biological functions of these 3 genes. The results of our research might reveal novel targets and strategies for the diagnosis and treatment of colon cancer, as well as provide a deeper insight into the pathogenesis of colon cancer, which would inform further research.

## Materials and methods

### Analysis of *SERPINE1* expression among different cancers and clinical features of colon cancer

Expression of the target gene *SERPINE1* in various cancers was analyzed through the TIMER database (http://cistrome.dfci.harvard.edu/TIMER/). The expression differences were examined by Wilcoxon method. Based on the starBase database (http://starbase.sysu.edu.cn/index.php) and the UALCAN database (http://ualcan.path.uab.edu/analysis.html), *SERPINE1* gene expression in Colon Adenocarcinoma (COAD) and normal tissue samples was detected. Detection of *SERPINE1* expression in colon cancer patients with various clinical features (age, gender, nodal metastasis status, race, stages, subtypes and weight) was performed using analysis of variance. All database parameters were set by default.

### Clinical samples

This study included 15 patients with colon cancer who underwent one-stage operation in Tangshan Central Hospital from May, 2020, to March, 2021. All patients included in the study had not received chemotherapy or radiotherapy before resection, and adjacent tissue and tumor tissue samples were immediately frozen at -80 ℃ until analysis. The study was approved by the Medical Ethics Committee of Tangshan Central Hospital, and participants proffered written informed consent for research purposes and publication.

### qRT-PCR detection

Total RNA was extracted from adjacent and tumor tissues utilizing Trizol (Invitrogen, USA). RNA was reversely transcribed into cDNA by PrimeScript RT kit (Takara, Japan). Gene expression in adjacent and tumor tissues was assessed utilizing miScript SYBR Green PCR Kit (Qiagen, Germany), and qRT-PCR analysis was done on 7500 Real-time PCR System (Applied Biosystems, USA). Beta-actin was utilized as an internal reference for mRNA. Relative expression levels of mRNA were calculated using the 2^−ΔΔCT^ method. Primers were as follows:

SERPINE1: Forward primer5’-GCAAGGCACCTCTGAGAACT-3’.

Reverse primer5’-GGGTGAGAAAACCACGTTGC-3’.

Beta-actin: Forward: 5’-ACAGAGCCTCGCCTTTGCC-3’.

Reverse: 5’-TGGCCATCTCTTGCTCGAAG-3’.

### Evaluation of the relationship between *SERPINE1* expression and patient survival

To assess prognostic value of *SERPINE1* for colon cancer, we used R package “survival” [14] for survival analysis to explore the impact of *SERPINE1* expression on the survival time of patients with different clinical phenotypes. First, patients were divided into high and low expression groups according to *SERPINE1* expression. Then the survival curve was plotted by Kaplan Meier (K–M) method, and compared by log-rank test. *P* value < 0.05 was regarded as significant.

### GSEA analysis

The LinkFinder module of the LinkedOmics (http://www.linkedomics.org/login.php) was utilized to analyze the co-expressed genes of *SERPINE1* based on TCGA-COAD cohort (*n* = 533), and Pearson correlation coefficient was calculated to evaluate the correlation between gene expression (false discovery rate [FDR < 0.01]). Then, the LinkInterpreter module was used to perform GSEA on the LinkFinder calculation results: First, the obtained LinkFinder data were labeled and sorted, and then GSEA was performed for Gene Ontology (GO) annotation, Kyoto Encyclopedia of Genes and Genomes (KEGG) pathway enrichment, kinase target enrichment, miRNA target enrichment and transcription factor target enrichment. Finally, the kinase targets, miRNA targets, transcription factor targets and corresponding signaling pathways related to *SERPINE1* were obtained. 500 simulations were performed. The top 10 genes with the most prominent FDR were selected for GO and KEGG analyses.

### GeneMANIA analysis

GeneMANIA (http://www.genemania.org) is a simple analysis software that can be used to establish gene regulatory networks and analyze the biological function of genes. gene regulatory networks with Kinase *MAPK1*, *miR-18a* and *SRF_Q* related genes were constructed, and relevant biological functions were analyzed based on GeneMANIA (http://www.genemania.org) database.

## Results

### Differential expression of *SERPINE1* in various cancers

**Fig. 1:**
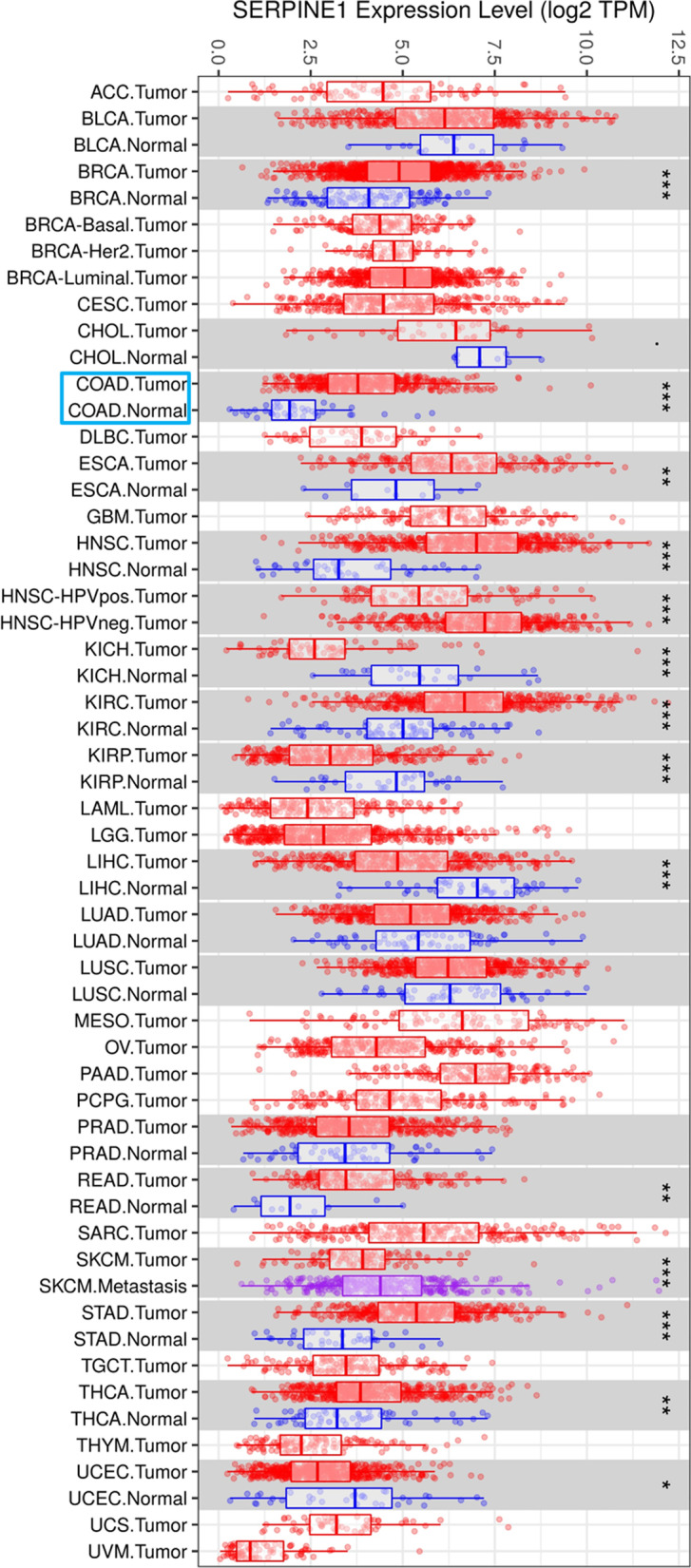
Expression of *SERPINE1* mRNA in different cancer types. *SERPINE1* levels in varying cancer tissues and normal tissues were analyzed through TIMER database. Red box: tumor sample; blue box: normal sample; purple box: metastasis sample; expression data of *SERPINE1* were normalized to log2 (TPM). Statistical analysis was conducted using Wilcoxon method. * *p* < 0.05, ** *p* < 0.01, *** *p* < 0.001

To understand the expression status of *SERPINE1* among various cancers, TIMER database was employed to analyze the expression of *SERPINE1* in various cancer tissues and the corresponding normal tissues. The results demonstrated that *SERPINE1* was dramatically highly expressed in breast cancer, colon cancer, esophageal cancer, head and neck squamous cell carcinoma, clear cell renal cell carcinoma, gastric cancer, and thyroid cancer tissue, while prominently lowly expressed in renal chromophobe cell carcinoma, renal papillary cell carcinoma, hepatocellular carcinoma, and endometrial cancer tissue (Fig. 1). Based on the analysis above, we selected colon cancer in which *SERPINE1* was highly expressed.

### SERPINE1 expression in colon cancer

**Fig. 2:**
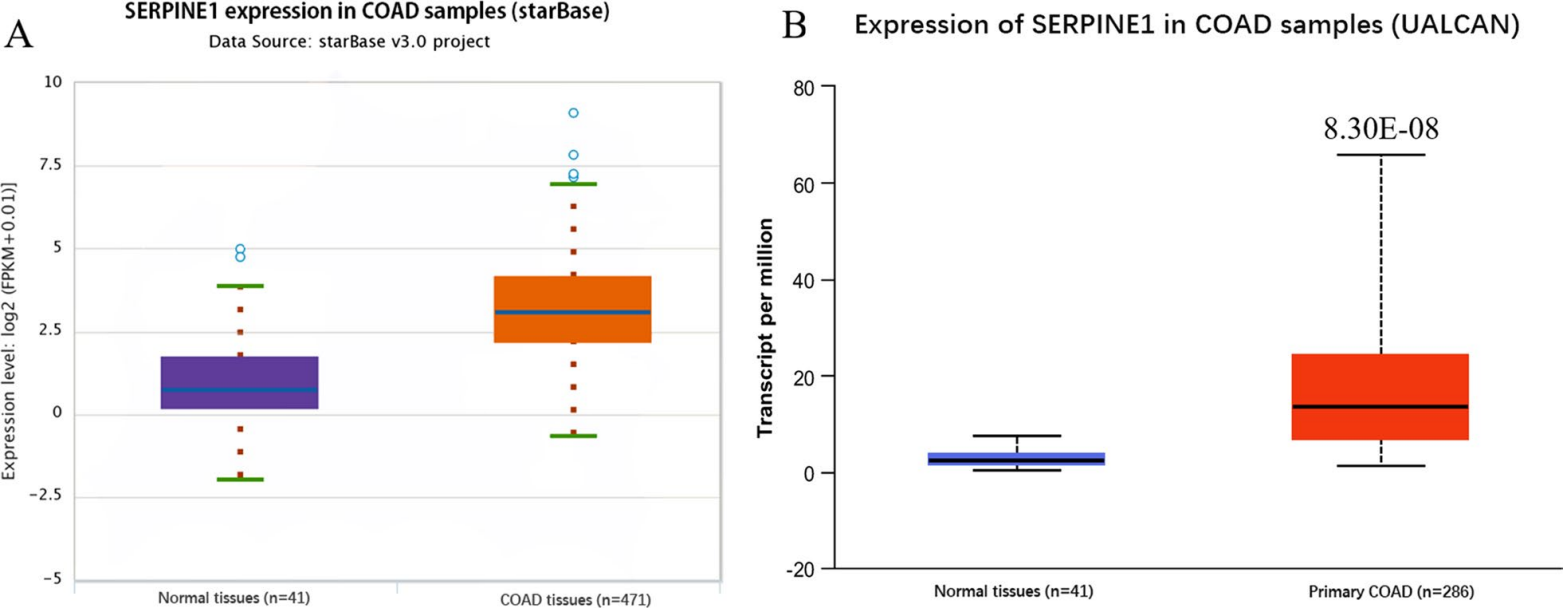
Expression of *SERPINE1* mRNA in colon cancer. **A** Analysis results of *SERPINE1* expression in starBase database; orange box represents tumor tissue (*n* = 471); purple box represents normal tissue (*n* = 41); expression data were normalized to log2 (FPKM + 0.01); **B** Analysis results of *SERPINE1* expression in UALCAN database (normal: *n* = 41; tumor: *n* = 286); expression data were normalized to TPM. Statistical analysis was conducted using Student’s *t*-test (*p* < 0.05 indicates a significant difference)

**Fig. 3:**
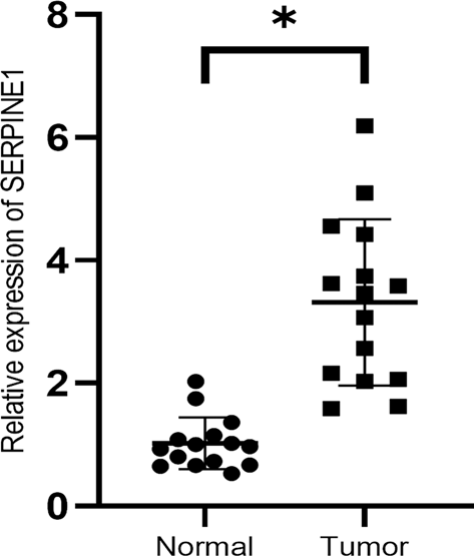
*SERPINE1* level in clinical tissues. Analysis of *SERPINE1* expression in 15 pairs of tumor tissues and adjacent tissues from patients with colon cancer. Statistical analysis was conducted using Student’s *t*-test. * *p* < 0.05

**Fig. 4:**
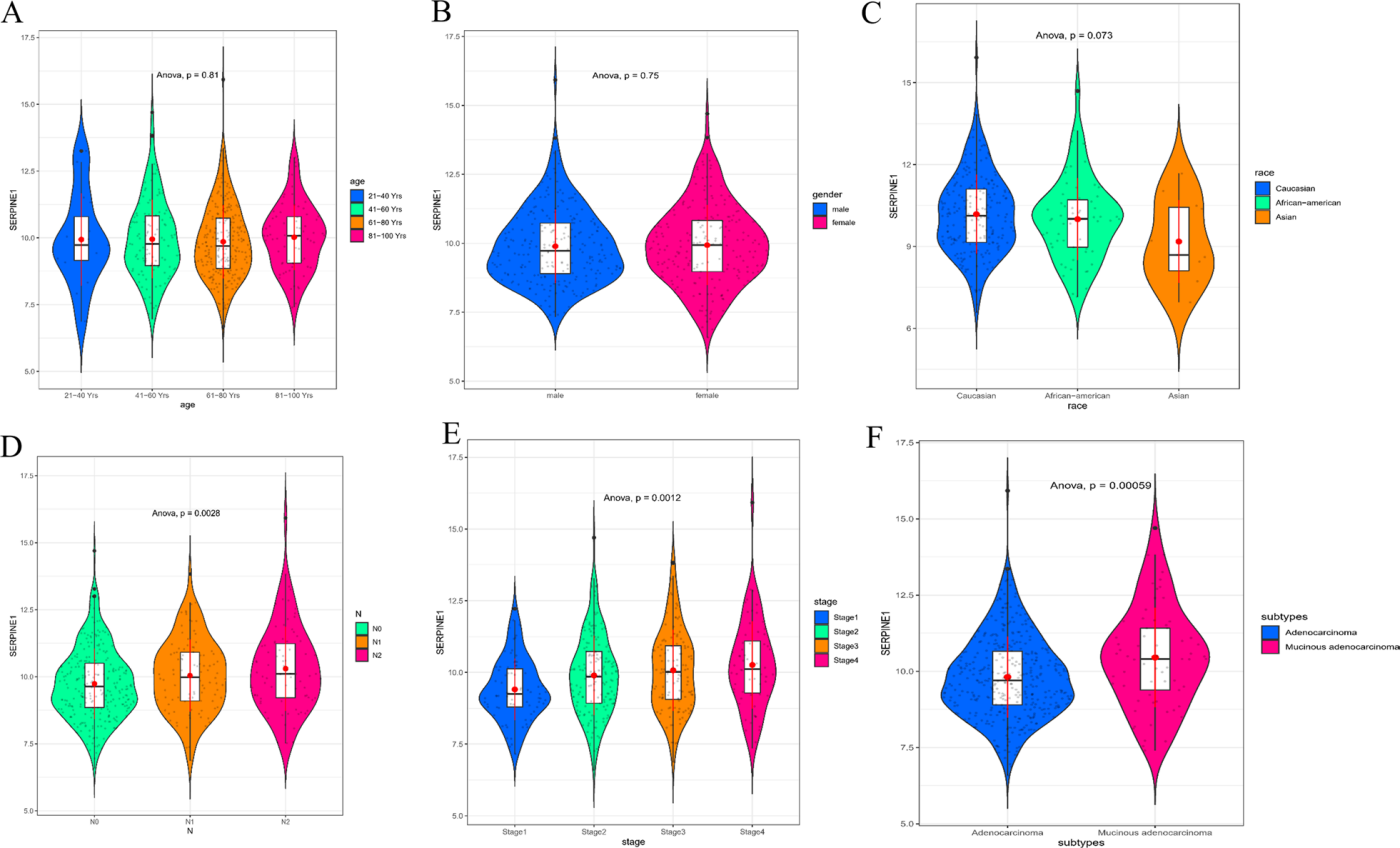
Association between *SERPINE1* expression and clinical characteristic. **A** Association between *SERPINE1* expression and patient’s age as analyzed by analysis of variance (21–40 Yrs: *n* = 12; 41–60 Yrs: n = 90; 61–80 Yrs: *n* = 149; 81–100 Yrs: *n* = 32); **B** Association between *SERPINE1* expression and patient’s gender as analyzed by analysis of variance (male: *n* = 156; female: *n* = 127); **C** Association between *SERPINE1* expression and patient’s race as analyzed by analysis of variance (Caucasian: *n* = 193; African-American: *n* = 55; Asian: *n* = 11); **D** Association between *SERPINE1* expression and patient’s nodal metastasis status as analyzed by analysis of variance (N0: *n* = 166; N1: *n* = 70; N2: *n* = 47); **E** Association between *SERPINE1* expression and patient’s cancer stage as analyzed by analysis of variance (stage1: *n* = 45; stage2: *n* = 110; stage3: *n* = 80; stage4: *n* = 39); **F** Association between *SERPINE1* expression and patient’s histological subtype as analyzed by analysis of variance (adenocarcinoma: *n* = 243; mucinous adenocarcinoma: *n* = 37) (*p* < 0.05 indicates a significant difference)

To comprehensively examine the expression condition of *SERPINE1* in colon cancer, gene expression of *SERPINE1* was further verified in the starBase and UALCAN databases. Consistently, the results of the expression analyses illustrated that *SERPINE1* was highly expressed in colon cancer tissue (*n* = 471/286) in contrast with the normal tissue (*n* = 41) (Fig. 2A–B). To validate *SERPINE1* level in clinical tissues, 15 pairs of tumor tissues and adjacent tissues were excised from colorectal cancer patients and compared *SERPINE1* expression. The findings presented that *SERPINE1* was highly expressed in cancer tissues in contrast with adjacent tissues (Fig. 3). Afterwards, the relationship analyses between the expression of *SERPINE1* and clinical phenotypes in colon cancer were subsequently conducted. As shown in Fig. 4A–F, the results illustrated that *SERPINE1* expression was associated with the malignant degree and clinical features (nodal metastasis status, stages, subtypes) of the colon cancer patients.

**Fig. 5:**
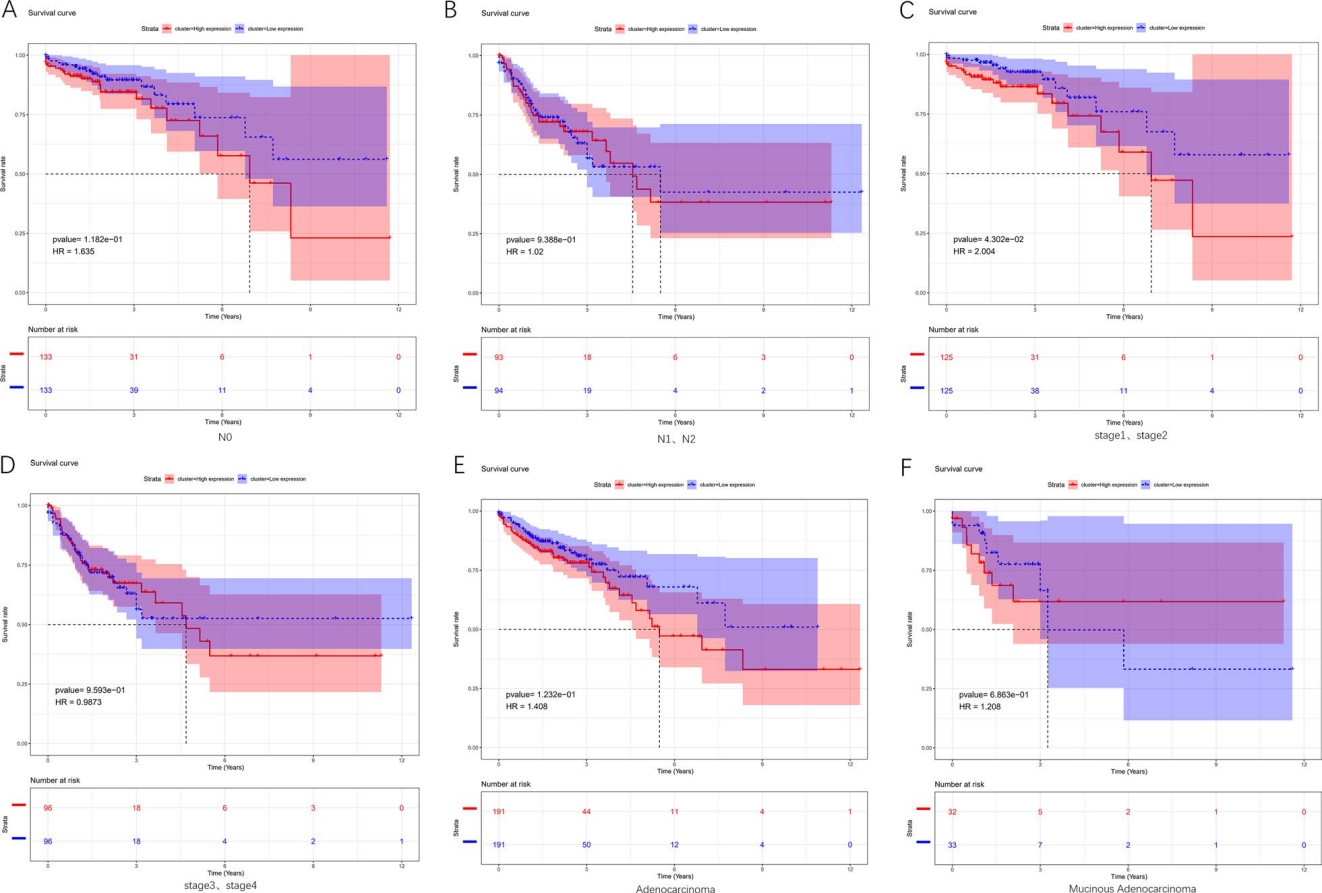
Association between *SERPINE1* level and colon cancer patients’ prognoses. **A** Survival curves of patients in N0 stage with high/low *SERPINE1* expression; **B** Survival curves of patients in N1 stage and N2 stage with high/low *SERPINE1* expression; **C** Survival curves of patients in stage1 and stage2 with high/low *SERPINE1* expression; **D** Survival curves of patients in stage3 and stage4 with high/low *SERPINE1* expression; **E** Survival curves of Adenocarcinoma patients with high/low *SERPINE1* expression; **F** Survival curves of Mucinous Adenocarcinoma patients with high/low *SERPINE1* expression. K–M method was used for survival analysis and drawing the survival curves, and difference among patients’ subgroups was calculated by log-rank test (*p* value < 0.05 indicates a significant difference)

To further explore the impact of clinical phenotypes and *SERPINE1* expression on the prognosis of colon cancer patients, survival curves of various clinical phenotypes were plotted. The results presented that survival time of patients in stage1 and those in stage2 was longer in the low expression group of *SERPINE1*. But other clinical phenotypes in the high and low *SERPINE1* expression groups were not strongly associated with patient’s survival (Fig. 5A–F). All of these illustrated that *SERPINE1* expression was associated with specific stages of cancer and prognoses of colon cancer patients.

### Functional enrichment analysis of *SERPINE1* co-expressed genes

**Fig. 6:**
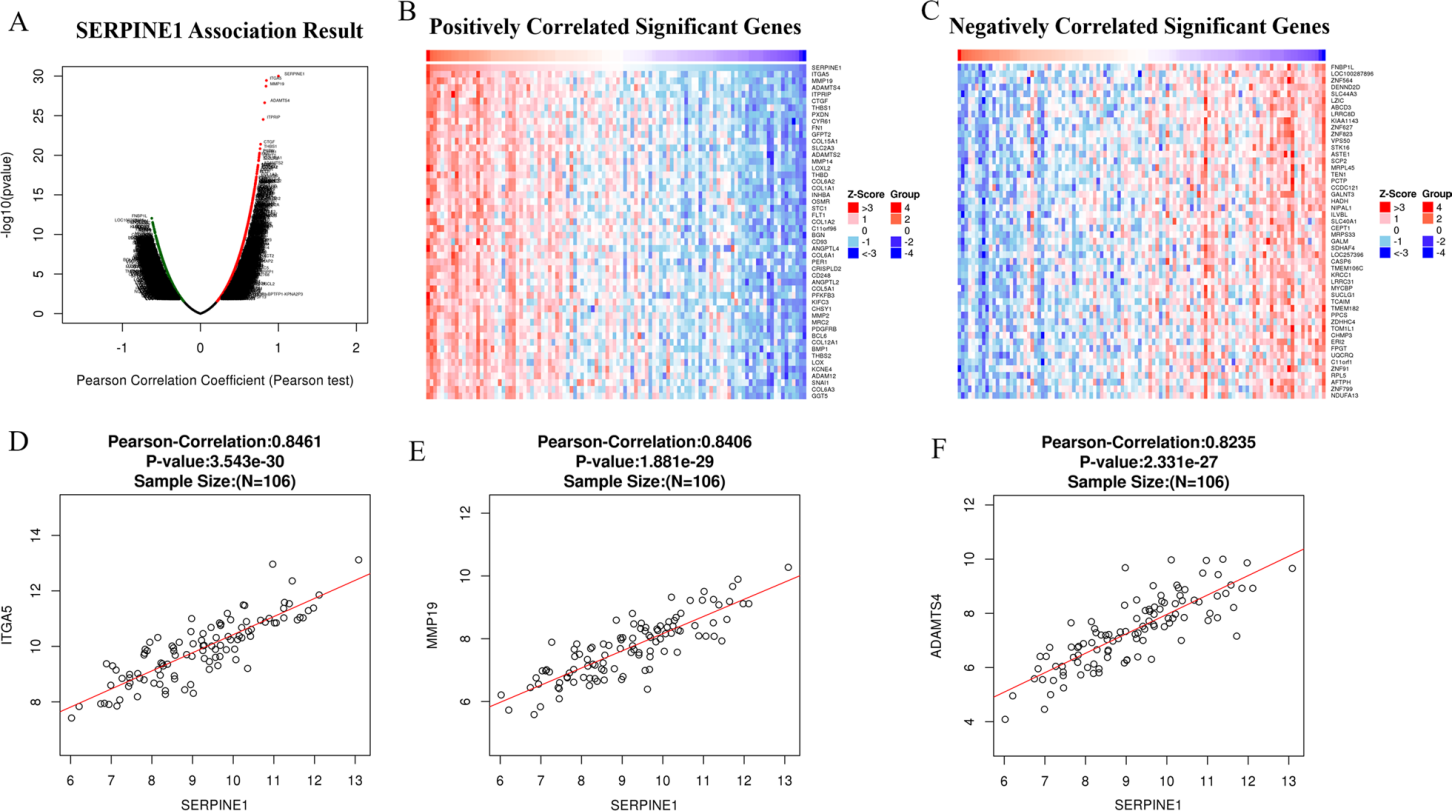
Relationship between *SERPINE1* and associated genes. **A** Correlation analysis of *SERPINE1* in TCGA-COAD (TCGA-Colon Adenocarcinoma); green dots represent downregulated genes; red dots represent upregulated genes; **B–C** Heatmap of the top 50 genes substantially positively/negatively associated with *SERPINE1*; Z-score value gradually decreases from red to blue; **D–F** *ITGA5*, *MMP19* and *ADAMTS4* with the highest correlation coefficient with key co-expressed genes of *SERPINE1* as calculated by Pearson correlation analysis (*p* value < 0.05 indicates a significant difference)

The LinkFinder module of the LinkedOmics database was utilized to perform correlation analysis based on the 106 colon cancer patients in TCGA database. The results of correlation analysis exhibited that a total of 5,407 genes were markedly correlated with *SERPINE1*, with 2,377 substantially positively correlated and 3,030 substantially negatively correlated (Fig. 6A). The Top 50 genes that were conspicuously positively/negatively correlated with *SERPINE1* were shown in heat maps in Fig. 6B–C. As can be seen from Fig. 6D–F, the three genes most significantly correlated to *SERPINE1* expression were *ITGA5*, *MMP19*, and *ADAMTS4*.

**Fig. 7:**
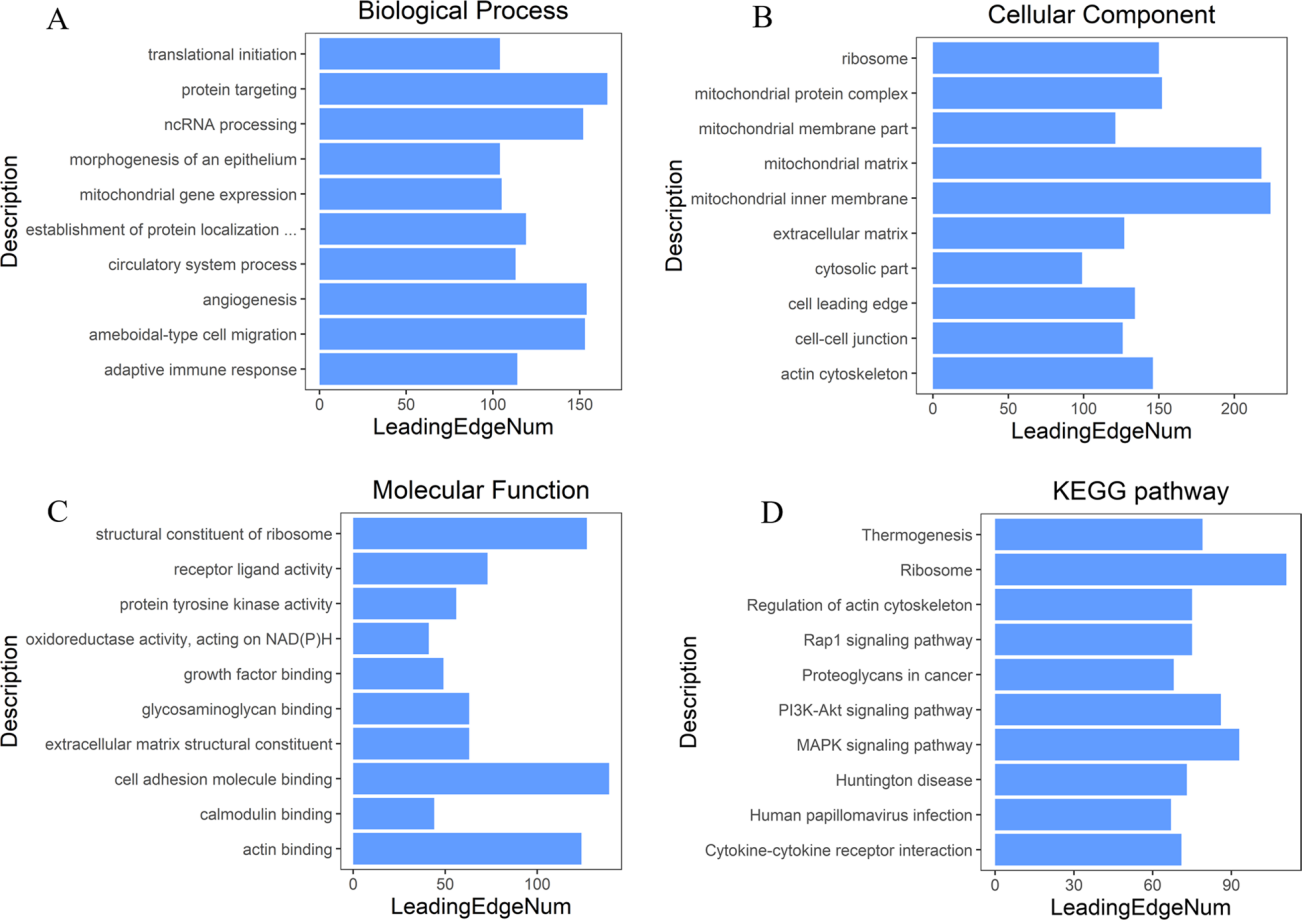
GSEA enrichment results for *SERPINE1* associated genes. **A–C** GO enrichment analysis of 10 *SERPINE1*-associated genes; **D** KEGG enrichment analysis of 10 *SERPINE1*-associated genes

Based on the top 10 genes significantly associated with *SERPINE1*, GO and KEGG analyses were employed to predict the potentially involved biofunctions and signaling pathways. GO enrichment analysis uncovered that changes in the expression of *SERPINE1* mainly affected biological processes such as translational initiation, protein targeting, ncRNA processing, which was mainly correlated with cellular components including ribosome, cell structures related to mitochondria and cell adhesion, as well as relevant molecular functions such as receptor ligand activity, protein tyrosine kinase activity (Fig. 7A–C). The results of KEGG enrichment analysis indicated that changes in *SERPINE1* expression mainly affected Rap1\PI3K-Akt and MAPK signaling pathways (Fig. 7D).

### Constructing co-expression networks based on *SERPINE1*-associated kinase, miRNA, and transcription factor

To further explore the potential targets of *SERPINE1* in colon cancer, *SERPINE1* networks for kinase, miRNA and transcription factor targets were analyzed by GSEA. The results signified that kinase targets were mainly related to *MAPK1*, *SRC*, *AKT1*, *FYN* and *MAPK8* kinase-related signaling pathways. The miRNA-target network was mainly related to *miR-18a*, *miR-29a*, *miR-29b*, *miR-29c*, *miR-24*, *miR-138* and *miR-9*. The transcription factor-target regulatory network was mainly related to several transcription factors such as *SRF-Q6*, *ZIC1*, *ZIC3*, *NF-κB* and *SRF-C* (Table 1).

**Table 1:** Constructing *SERPINE1* regulatory networks for kinase, miRNA, and transcription factor targets in colon cancer

Enriched category	GeneSet	FDR	LeadingEdgeNum
Kinase target	Kinase_MAPK1	0	84
	Kinase_SRC	0	71
	Kinase_AKT1	0	67
	Kinase_FYN	0	28
	Kinase_MAPK8	7.89E-04	41
miRNA Target	AGGGCAG, miR-18a	0	50
	TGGTGCT, miR-29a, miR-29b, miR-29c	0	122
	CTGAGCC, miR-24	0	68
	CACCAGC, miR-138	0.000421	68
	ACCAAAG, miR-9	0.000449	137
Transcription factor Target	V$SRF_Q6	0	90
	V$ZIC1_01	0	84
	V$ZIC3_01	0	79
	V$NFKAPPAB_01	0	78
	V$SRF_C	0	76

**Fig. 8:**
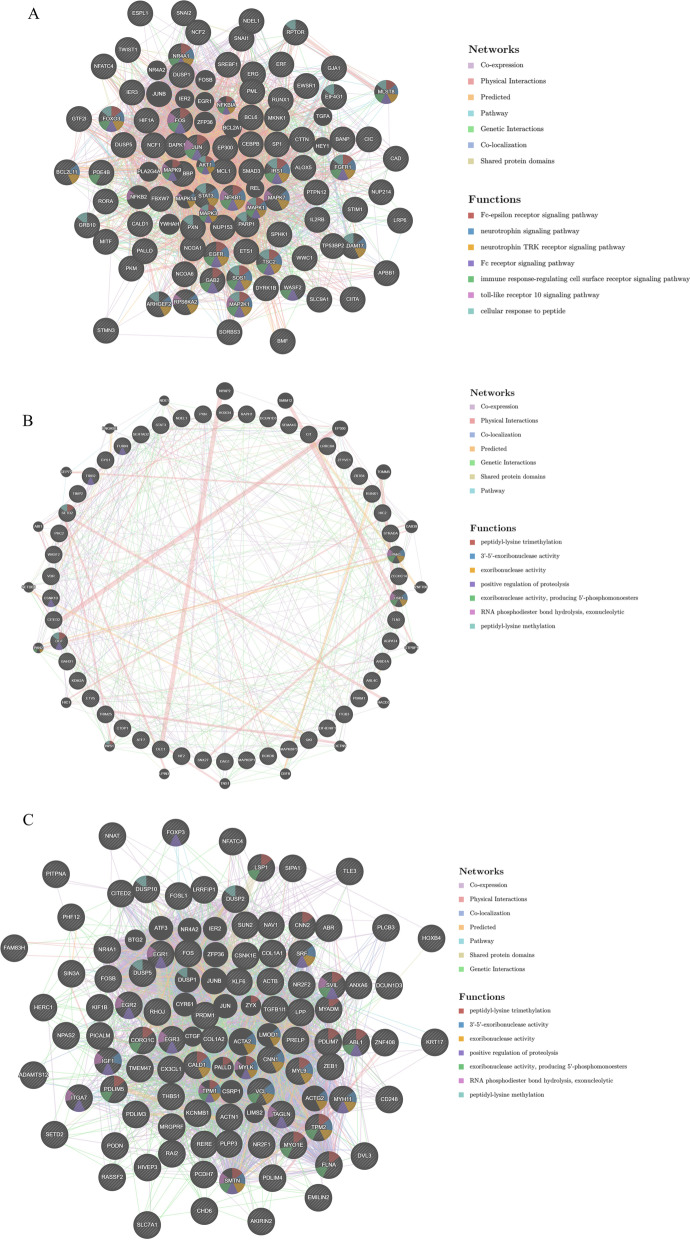
Analysis results of GeneMANIA. **A** A gene regulatory network with kinase *MAPK1* related genes constructed based on the GeneMANIA database; **B** A gene regulatory network with *miR-18a* related genes constructed based on the GeneMANIA database; **C** A gene regulatory network with *SRF-Q6* related genes constructed based on the GeneMANIA database

In the GeneMANIA database, genes associated with kinase *MAPK1*, *miR-18a* and *SRF-Q6* were used to construct a regulatory network. The results revealed that *MAPK1* was mainly involved in neurotrophin signaling pathway, Fc receptor signaling pathway, and toll-like receptor 10 signaling pathway, etc. (Fig. 8A). *MiR-18a* mainly participated in 3’-5’-exoribonuclease activity, positive regulation of proteolysis, exoribonuclease activity, producing 5’-phosphomonoesters, RNA phosphodiester bond hydrolysis, exonucleolytic and other biological processes (Fig. 8B). The transcription factor *SRF-Q6* was mainly involved in the modulation of actin cytoskeleton, actin-binding, *MAP* kinase tyrosine/serine/threonine phosphatase activity (Fig. 8C). Together, these findings suggested that in colon cancer, *SERPINE1* may be involved in signaling pathways or cell functions of *MAPK1*, *MiR-18a* and *SRF-Q6*. Changes in expression of these genes may also cause alternation in specific cell functions.

## Discussion

*SERPINE1* (*PAI-1*) is proven by numerous studies to be a pivotal gene influencing the occurrence and progression of cancer. Current research believes that *SERPINE1* can catalyze the degradation of basement membrane and ECM, making it easier for cancer cells to invade surrounding normal tissue and promote the progression of cancer [15]. In this study, through the analysis of TIMER database, it was found that *SERPINE1* had obvious differential expression in various cancers, and the most significant differential expression was present in colon cancer. Colon cancer as a common type of cancer attracted a large number of studies on changes in its gene expression. For example, changes in *FGFRL1* gene can promote the occurrence and progression of colon cancer [16]. *WISP* can extend the survival time of patients through the WISP2/β-catenin pathway [17]. Based on the analysis of the relevant data from The Human Protein Atlas and the UALCAN database, this study found that the expression of *SERPINE1* in colon cancer tissue was significantly up-regulated, demonstrating that *SERPINE1* may be a key gene involved in development and progression of colon cancer. This study also explored the expression of *SERPINE1* in patients with various clinical characteristics in the UALCAN database. The findings indicated that the expression of *SERPINE1* was generally higher in patients with various clinical characteristics than that in healthy individuals. By comparing *SERPINE1* with tumor-associated factors, it was revealed that *SERPINE1* level in stage1 of colorectal cancer substantially varied from other 3 stages, and *SERPINE1* was related to TMN stages of CRC [18]. Furthermore, the differences in survival time of patients with high/low *SERPINE1* expression were also analyzed in combination with clinical characteristics, and the results showed that the survival time of patients with high *SERPINE1* expression was dramatically shorter. Li et al*.* [19] reported that *SERPINE1* is highly expressed in gastric adenocarcinoma and is dramatically implicated in patients’ prognoses. Forced expression of *SERPINE1* (PAI-1) is noticeably related to shorter survival of glioblastoma patients [20]. Another study indicated that gastric cancer patients with high *SERPINE1* expression have shorter OS than those with low *SERPINE1* expression [21].

The top 10 genes significantly associated with *SERPINE1* were assessed by GO and KEGG to predict the potential biological functions and signaling pathways involved. GO enrichment analysis reported that changes in *SERPINE1* expression mainly affected cellular components related to ribosomes, cell structure and cell adhesion, receptor ligand activity, and protein tyrosine kinase activity. KEGG enrichment analysis presented that alternations in *SERPINE1* expression mainly affected Rap1\PI3K-Akt and MAPK signaling pathways. Zhang et al*.* [22] reported that protein tyrosine kinase is poorly expressed in breast cancer cells and represses invasion and metastasis of breast cancer cells. Xiong et al*.* [23] found that 6 differentially expressed miRNA target genes in osteosarcoma involved in the PI3K-Akt signaling pathway. Forced expression of *ART3* stimulates proliferation of triple-negative breast cancer cells and modulates triple-negative breast carcinogenesis via activation of Akt and ERK pathways [24].

Subsequently, it was found through the LinkedOmics database that there were a great number of genes associated with the expression of *SERPINE1*, wherein the expression of *ITGA5*, *MMP19* and *ADAMTS4* was the most pronouncedly related to the expression of *SERPINE1*. *ITGA5* is integrin subunit α5, and there are little published data on proving that relationship between *SERPINE1* and the expression of *ITGA5*, while it is reported that overexpression of *ITGA5* can promote the metastasis and spread of colon cancer [25]. *MMP19* (matrix metallopeptidase 19) is related to epithelial cell proliferation, enterocyte migration and poor prognosis [26, 27]. *ADAMTS4* is highly expressed in colon cancer cells, and research suggested that *ADAMTS4* can promote cancer progression through macrophages [28, 29]. *SERPINE1* is notably positively correlated with these three genes, that is, *SERPINE1* and these three genes may jointly played important roles in the progression of colon cancer.

Exploring biological pathways is an important method for cancer research. Studies found that biological pathways such as *MAPK* pathway and *NF-κB* pathway can affect the pathogenesis of colon cancer [30, 31]. Our research found that *SERPINE1* might affect the pathogenesis of colon cancer by influencing cell adhesion, kinase activation and other pathways, and thus we further studied the kinases, miRNAs and transcription factors related to *SERPINE1*. Kinases play a pivotal role in the pathogenesis of cancer and research suggested that the activation of the tyrosine kinase pathway can stimulate the pathogenesis of colon cancer [32]. This study found that the *MAPK1* kinase pathway and *SRC* kinase were important causes of colon cancer, while *MAPK1* is a common cancer-promoting pathway that is believed to promote the progression of prostate cancer and breast cancer [33, 34]. In addition, *SRC* is also a common cancer-promoting pathway, and it can promote the occurrence of breast cancer by inducing mitochondrial dysfunction [35]. Besides, miRNAs are also common cancer-related regulatory factors. MiRNAs such as *miR-770‑5p* and *miR-200* family are found to be related to the risk of colon cancer [36, 37]. Here, *miR-18a* and *miR-29a* were found in the *SERPINE1*-miRNA regulatory network. *MiR-18a* can affect the onset of colon cancer through the Cdc42/filopodia pathway, and *miR-29a* can affect the onset of colon cancer by regulating *b-3p-COL5A1* [38, 39]. In addition to miRNA, transcription factors are also important regulators that affect pathogenesis. Research suggested that the aberrant expression of transcription factors such as *KLF14* and *SOX2* in colon cancer can affect the occurrence and progression of colon cancer by affecting the expression of downstream genes [40, 41]. This study found that *SERPINE1* was associated with *SRF-Q6* and *ZIC*, which are serum response factor and zinc finger protein, respectively. Research suggested that *SRF-Q6* can affect the occurrence of cancer through the SRF/MRTF pathway while *ZIC* can induce colon cancer by influencing the expression of *GLUT1*^42^, ^43^. After conducting the above research, GeneMINIA was used to establish regulatory networks. The results demonstrated that *MAPK1* was mainly involved in immune-related pathways, *miR-18a* was mainly involved in serotonin methylation-related pathways, and *SRF-Q6* was mainly involved in actin-related pathways.

In summary, this study dived into *SERPINE1* (PAI-1) through bioinformatics methods and explored the genes and pathways related to *SERPINE1* expression. *SERPINE1* expression was found dramatically related to the expression of *ITGA5*, *MMP19*, and *ADAMTS4*, and affected biological pathways such as RNA transcription, cell adhesion, and kinase activation. This study also identified regulatory networks of *SERPINE1* with kinases, miRNAs, and transcription factors through GSEA, such as *MAPK1* kinase, *miR-18a*, and transcription factor *SRF-Q6*, etc. The contribution of this study is to fully explore the genes and pathways that might interact with *SERPINE1*. However, this study has the following two limitations. First, this paper is mainly based on bioinformatics methods for analysis, lacking of cell or clinical experiments for verification. Additionally, we conducted bioinformatics analysis directly based on the databases without special treatment, and there may be differences in sample quality, leading to some deviations in the results. We will design relevant experiments in the follow-up study to verify the clinical significance of *SERPINE1*, and will look for more sample data for optimization to reduce the deviation of results.

## Data Availability

The data used to support the findings of this study are included within the article. The data and materials in the current study are available from the corresponding author on reasonable request.
